# Excellent Hole Mobility and Out–of–Plane Piezoelectricity in X–Penta–Graphene (X = Si or Ge) with Poisson’s Ratio Inversion

**DOI:** 10.3390/nano14161358

**Published:** 2024-08-17

**Authors:** Sitong Liu, Xiao Shang, Xizhe Liu, Xiaochun Wang, Fuchun Liu, Jun Zhang

**Affiliations:** 1 Institute of Atomic and Molecular Physics, Jilin University, Changchun 130012, China; sitong22@mails.jlu.edu.cn (S.L.); shangxiao22@mails.jlu.edu.cn (X.S.); liu_xizhe@jlu.edu.cn (X.L.); 2 School of Physics Science and Information Technology, Liaocheng University, Liaocheng 252000, China; wangxiaochun@tsinghua.org.cn

**Keywords:** first–principles calculation, 2D materials, negative Poisson’s ratio, piezoelectricity, carrier mobility

## Abstract

Recently, the application of two–dimensional (2D) piezoelectric materials has been seriously hindered because most of them possess only in–plane piezoelectricity but lack out–of–plane piezoelectricity. In this work, using first–principles calculation, by atomic substitution of penta–graphene (PG) with tiny out–of–plane piezoelectricity, we design and predict stable 2D X–PG (X = Si or Ge) semiconductors with excellent in–plane and out–of–plane piezoelectricity and extremely high in–plane hole mobility. Among them, Ge–PG exhibits better performance in all aspects with an in–plane strain piezoelectric coefficient *d*_11_ = 8.43 pm/V, an out–of–plane strain piezoelectric coefficient *d*_33_ = −3.63 pm/V, and in–plane hole mobility *μ_h_* = 57.33 × 10^3^ cm^2^ V^−1^ s^−1^. By doping Si and Ge atoms, the negative Poisson’s ratio of PG approaches zero and reaches a positive value, which is due to the gradual weakening of the structure’s mechanical strength. The bandgaps of Si–PG (0.78 eV) and Ge–PG (0.89 eV) are much smaller than that of PG (2.20 eV), by 2.82 and 2.47 times, respectively. This indicates that the substitution of X atoms can regulate the bandgap of PG. Importantly, the physical mechanism of the out–of–plane piezoelectricity of these monolayers is revealed. The super–dipole–moment effect proposed in the previous work is proved to exist in PG and X–PG, i.e., it is proved that their out–of–plane piezoelectric stress coefficient *e*_33_ increases with the super–dipole–moment. The *e*_33_–induced polarization direction is also consistent with the super–dipole–moment direction. X–PG is predicted to have prominent potential for nanodevices applied as electromechanical coupling systems: wearable, ultra–thin devices; high–speed electronic transmission devices; and so on.

## 1. Introduction

Penta–graphene (PG) is a two–dimensional (2D) material with the singular elastic property of a negative Poisson’s ratio (*ν* = −0.080) [1]. Compared to ordinary materials, 2D materials with a negative Poisson’s ratio expand (contract) rather than contract (expand) along the *y*–axis when subjected to *x*–axial tension (compression). Two–dimensional materials with an NPR like black phosphorus (*ν_y_* = −0.027) [2,3] and B_4_N (*ν_x_* = −0.018 and *ν_y_* =−0.032) [4] can achieve unusually advantageous material properties, such as high indentation resistance and high shear resistance, which have received attention in fields like damping material and nutrition research [5]. The advantage of 2D piezoelectric materials lies in their expansive in–plane area. When significant accumulation of polarized charges occurs on their surfaces, the application value of 2D piezoelectric materials becomes remarkably significant. This requirement highlights the necessity for these materials to possess excellent out–of–plane piezoelectricity. Unfortunately, the majority of 2D piezoelectric materials demonstrate solely in–plane piezoelectricity, attributed to their complete symmetry in the vertical direction (perpendicular to the material plane). This limitation stands as a significant obstacle impeding the widespread utilization of 2D piezoelectric materials [6,7]. Two–dimensional materials with excellent out–of–plane piezoelectricity [8,9,10,11,12] can be used in nanogenerators, energy harvesting and storage devices, and biomedical fields [13,14,15]. Two–dimensional ultra–thin semiconductors, such as monolayer *α*–In_2_Se_3_ with a large out–of–plane piezoelectric coefficient *d*_33_ = 0.34 pm/V, are widely used for sensors, actuators, electronics, and energy conversion [16]. In addition, the high carrier mobility of 2D materials is conducive to generating high–performance flexible electronic devices [17] and also has good application value in electronic devices [18]. Two–dimensional materials, such as graphene [19], molybdenum disulfide [20], etc., are very suitable for manufacturing high–performance electronic devices due to their excellent electrical properties and ultra–high carrier mobility. For example, they can be applied to field–effect transistors (FETs), logic gate circuits, etc., significantly improving the speed and efficiency of devices. Two–dimensional materials with high mobility help reduce the transistor size and improve the performance and integration of integrated circuits, thus driving further miniaturization and functionalization of electronic products. In the field of energy, 2D materials with high mobility, as per the light absorption layer or the transport layer of solar cells, can improve the conversion efficiency and stability of solar cells. They are able to collect and use solar energy more efficiently and convert it into electricity. Two–dimensional materials with high mobility also have potential applications in energy storage devices such as supercapacitors and lithium–ion batteries. Their high surface area and excellent electrical properties help to improve the energy density and power density of energy storage devices. PG has low thermal conductivity [21] and ferroelectric and catalytic properties, but its out–of–plane piezoelectricity is extremely weak (*d*_36_ = −0.065 pm/V) [22] due to its extremely high symmetry (space point group D_2d_). Therefore, we modified PG to break its perfect vertical symmetry and designed two X–PG monolayers (X = Si or Ge) with excellent in–plane and out–of–plane piezoelectricity and extremely high in–plane hole mobility. The former has a negative Poisson’s ratio, while the latter has a positive Poisson’s ratio.

Zhang, H. N. [23] et al. found that the F–B–H monolayer in F–M–H (M = B, Al, and Ga) group–III(A) Janus hydrofluoride has the larger vertical piezoelectric coefficient and proposed a vertical piezoelectric mechanism such that the larger the electronegativity difference ratio between the atom layers, the better the vertical piezoelectric properties of the corresponding monolayer. Similarly, Li, Y. Q. [24] et al. defined the P–R mechanism in their explanation of vertical piezoelectric properties. In a word, the greater the amount of electron transfer, the more polarized charges will appear on the surface of 2D piezoelectric materials after the internal charge transfer induced by external stress, resulting in better corresponding vertical piezoelectric properties. However, the P–R mechanism is only currently applicable to three–atomic–layer molecular monolayers but not to molecular monolayers with more (>3) atomic layers. M. Noor–A–Alam et al. [25] explain well the large piezoelectricity in four–atomic–layer materials using *Z*_33_* Born effective charges (BECs), but they do not consider the contribution of the *Z*_33_* BECs and the location of all the atoms to the vertical piezoelectricity. Hence, the vertical piezoelectric physical mechanism of 2D piezoelectric materials remains to be further explored. Herein, the physical mechanism of the out–of–plane piezoelectricity of these monolayers is revealed. Using Born effective charges–centers and the super–dipole–moment concept, we have explained the internal physical mechanism dictating the difference in the out–of–plane piezoelectricity of PG and X–PG monolayers well.

## 2. Theoretical Methods

We conducted the first–principles calculations using the projector–augmented wave (PAW) method within the Vienna Ab initio Simulation Package (VASP) [26,27,28]. The exchange–dependent functional used in the Perdew–Burke–Ernzerhof (PBE) function forms the generalized gradient approximation (GGA) [29]. The plane wave energy cutoff is set to 520 eV. The fully relaxed structure with a 20 Å vacuum layer converges when the maximum force reaches 10^−3^ eV/Å, and the self–consistent field energy reaches 10^−6^ eV. The first Brillouin zone is sampled using an 8 × 8 × 1 Monkhorst–Pack *k*–point grid. The accurate band electronic structure for the PG monolayer is calculated by the Heyd–Scuseria–Ernzerhof (HSE06) hybrid function [30]. Following the above settings, for our calculation of PG (the data from previous studies are in brackets below), the lattice constant is 3.64 Å (3.63 Å) [31], the bandgap is 2.20 eV (2.22 eV) [32], the electronic band structure and the phonon spectrum of PG (see Figure S1 in the Supplementary Materials) are consistent with the previous study [22], the elastic coefficient *C*_11_ = *C*_22_ = 269.08 N/m (270.2 N/m) [33], Poisson’s ratio is −0.079 (−0.080) [1], and *d*_36_ = −0.063 pm/V (−0.065 pm/V) [34]. Our results for PG align very well with prior research, which confirms the accuracy of our first–principles calculations. The theory on piezoelectricity and deformation potential approximation in PG and X–PG monolayers is located in the Supplementary Materials.

## 3. Results and Discussion

**Figure 1:**
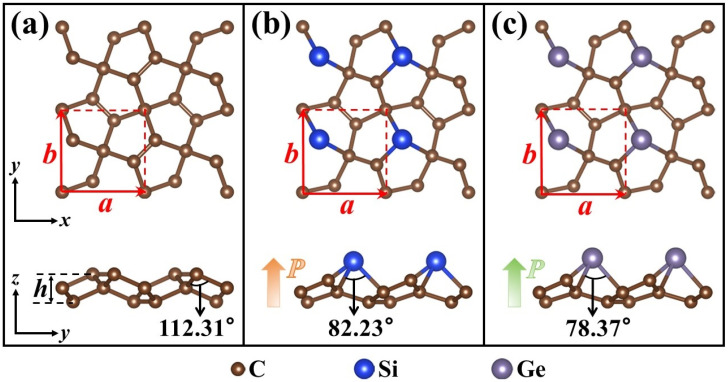
Top and side views of 2 × 2 supercells of (**a**) PG, (**b**) Si–PG, and (**c**) Ge–PG.

**Figure 2:**
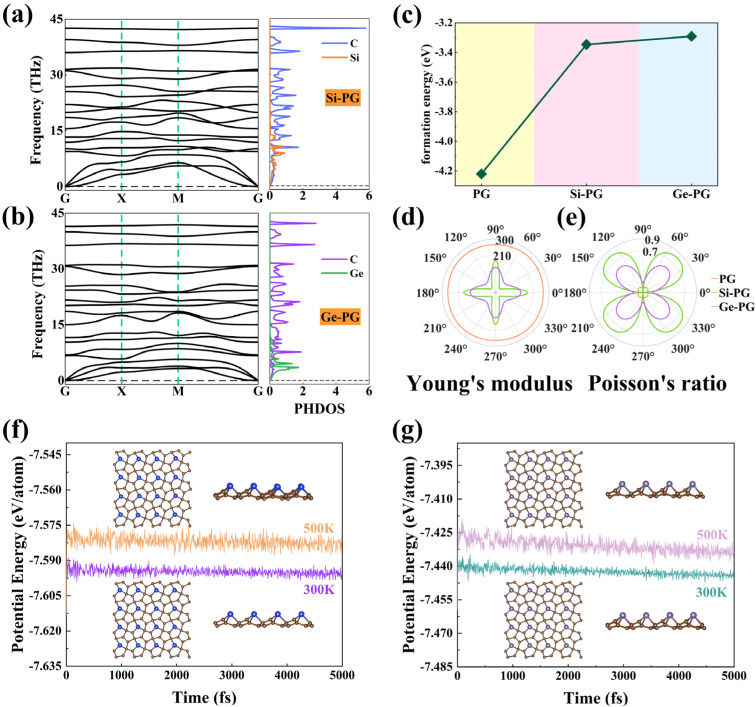
The phonon spectra with corresponding phonon density of states (PHDOS) of (**a**) Si–PG and (**b**) Ge–PG monolayers. (**c**) Formation energy; (**d**) Young’s modulus (in units of N/m); (**e**) Poisson’s ratio of PG and X–PG; (**f**,**g**) AIMD simulations of ground states of X–PG.

As shown in the top views of Figure 1, a C atom in the PG unit cell is substituted by an X atom to obtain X–PG monolayers. As shown in the side views of Figure 1b,c, the C atom substituted by an X atom in PG is one of the two C atoms with the largest *z*–axis coordinate. The relationship between C, Si, and Ge’s atomic radii (*r*_C_ (0.77 Å) < *r*_Si_ (1.17 Å) < *r*_Ge_ (1.22 Å)) determines the numerical relationship between the lattice constants *a* and the thicknesses *h* of PG and X–PG: *a*_PG_ (3.64 Å) < *a*_Si–PG_ (3.75 Å) < *a*_Ge–PG_ (3.76 Å), and *h*_PG_ (1.21 Å) < *h*_Si–PG_ (1.91 Å) < *h*_Ge–PG_ (2.05 Å) (as shown in Table 1). As shown in Figure 2a,b, the phonon frequencies of PG and X–PG are all positive in the Brillouin zone, which proves the dynamic stability of these monolayers. The phonon density of states (PHDOS) on the right corresponds to the corresponding phonon spectra. In X–PG, the high–frequency optical modes are caused by light C atoms, and the remaining low–frequency phonon modes are partially caused by heavy Si and Ge atoms [23,35]. The formation energy Δ*E* can be used to evaluate the possibility of obtaining an experimental synthesis material, which is calculated by [24]:

(1) $$△E=\frac{E_{tot}-n_{C}\times E_{C}-n_{Si/Ge}\times E_{Si/Ge}}{n_{tot}},$$

where *E_tot_* represents the total energy of the unit cells of PG and X–PG. *E_C_* and *E_Si/Ge_* represent the chemical potentials of C and Si/Ge atoms, respectively. *n_C_*, *n_Si/Ge_*, and *n_tot_* denote C and Si/Ge’s atomic numbers and the total atomic numbers in the unit cell, respectively. As shown in Figure 2c, the Δ*E* of PG = −4.22 eV, Si–PG = −3.35 eV, and Ge–PG = −3.29 eV, respectively. The negative formation energy value shows that these monolayers are thermodynamically stable. These things considered, in the NVT system, AIMD simulation with potential energy fluctuates slightly near a fixed value (Figure 2f,g), revealing the thermal stability of the ground state at 300 K and 500 K, respectively.

A discussion of the elastic coefficient tensor *C_kl_* with its subscripts and matrices can be seen in the Supplementary Materials. The *C_kl_* data for PG and X–PG are listed in Table 1. These monolayers all meet the Born–Huang criterion [36], *C*_11_*C*_22_ − *C*_12_^2^ > 0 and *C*_66_ > 0, which proves that PG and X–PG are mechanically stable. Since the lattice constants of PG and X–PG are the same along the *x* and *y* directions (*a* = *b*), their elastic constants *C*_11_ are equal to *C*_22_. This means that they have the same elastic properties in the *x* and *y* directions. Here, we focus on the Young’s modulus *Y_a_* (=*Y_b_*) and Poisson’s ratio ν*_a_* (=ν*_b_*) values of these monolayers along the *x* (*y*)–axis, where the subscripts *a* and *b* represent the angle between the direction of the external stress applied to the monolayer and the *x*–axis is at 0° and 90°, respectively. Then, *Y_a_* and ν*_a_* of these monolayers can be calculated by the following formulas:

(2) $$Y_{a}=Y(0^{{}^{\circ}})=\frac{C_{11}^{2}-C_{12}^{2}}{C_{11}},$$

(3) $$\nu_{a}=\nu (0^{{}^{\circ}})=\frac{C_{12}}{C_{11}},$$

Combining formula 3, as shown in Table 1, the negative Poisson’s ratios of PG and Si–PG are derived from their negative *C*_12_ values (−21.27 and −4.15 N/m), and the positive Poisson’s ratio of Ge–PG is caused by its positive *C*_12_ value (31.62 N/m). The *C*_12_ value of Si–PG is five times smaller than that of PG, which is the reason ν*_a_* of Si–PG is as small as −0.001. This indicates that the Si–PG monolayer will undergo an extremely small, almost non–expansion (contraction) response along the *y*–axis when subjected to external stretching (compression) along the *x*–axis. Therefore, the Si–PG monolayer is a desirable candidate for a 2D shock absorption material. As shown in the side views of Figure 1, the angle between the pre–replacement C atom’s two nearest neighboring C atoms and itself in PG decreases from 112.31° to the smaller angle between the Si and Ge atoms’ two nearest neighboring C atoms and themselves in the Si–PG (82.23°) and Ge–PG (78.37°) monolayers. The reduction in this bond angle contributes to the Poisson’s ratio inversion between PG, Si–PG, and Ge–PG. It manifests that the interactions between the atoms in Ge–PG become weaker, which is reflected in the higher lattice constant and thickness of Ge–PG than those of PG and Si–PG. The elastic coefficient *C*_11_ of Ge–PG (146.68 N/m) is smaller than that of PG (269.08 N/m) and Si–PG (185.60 N/m), which indicates that deformation occurs more easily in Ge–PG along the *x* (*y*)–axis. The structure of Ge–PG is more incompact, and its mechanical properties tend to be like those of conventional materials with positive Poisson’s ratios. Due to the high symmetry D_2h_ of the elemental carbon in PG, its Young’s modulus and Poisson’s ratio are isotropic, which is reflected in the perfect circulars in the polar coordinates in Figure 2d,e. Due to the substitution of X atoms for C atoms in the X–PG monolayers, their Young’s modulus and Poisson’s ratios are anisotropic and reach the maximum Young’s modulus values and minimum Poisson’s ratio values along the *x*/*y* axis (as shown in Table 1). *Y_a_* of Si–PG (178.41 N/m) and Ge–PG (142.17 N/m) is smaller than that of PG (267.40 N/m), which indicates that they are more flexible than PG in the *x*/*y* direction. As shown in Figure 2f,g, the AIMD simulation exhibits minor fluctuations around a fixed value, indicating the ground states of the X–PG monolayers possess thermal stability at 300 K and 500 K. Therefore, X–PG monolayers have potential as excellent flexible 2D materials.

**Figure 3:**
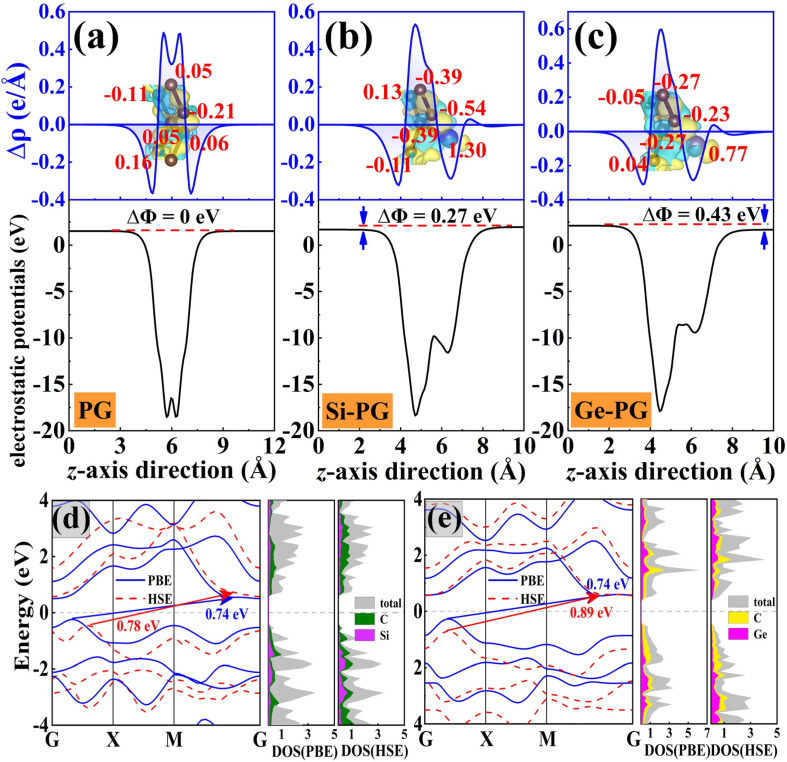
Planer–average charge density difference with Bader charge analysis (in red numbers) and electrostatic potentials of (**a**) PG, (**b**) Si–PG, and (**c**) Ge–PG monolayers. Band structures with the corresponding projected density of states (PDOS) of (**d**) Si–PG and (**e**) Ge–PG. The unit of PDOS is states/(eV • unit cell). The Fermi level is set to zero.

As shown in Figure 3a, the charge differential density and the electrostatic potential curves of PG along the *z*–axis appear as two symmetric curves due to the structural mirror–reversed symmetry with respect to the *xoy* plane. The electrostatic potential of the upper and lower surfaces of the PG monolayer is equal, so there is no vertical intrinsic polarization in PG. As shown in Figure 3b,c, the X–PG monolayers break the above mirror–reversed symmetries, so asymmetric charge transfer occurs within them. Since the electronegativity value of the Si (1.9) and Ge (2.01) atoms is smaller than that of the C atoms (2.55), both X atoms with positive Bader charge values lose electrons in each monolayer. The electronegativity value of the Si atom is smaller than that of the Ge atom, which means that Si atoms are more likely to lose electrons than Ge atoms. Therefore, the Si atom in Si–PG loses more charge than the Ge atom in Ge–PG and has a larger Bader charge value (1.30 |e|). In addition, the non–zero electrostatic potential differences between the upper and lower surfaces of X–PG monolayers are the source of their strong out–of–plane piezoelectricity. Ge–PG has a larger ΔΦ (0.43 eV) than Si–PG (0.27 eV), so Ge–PG also has stronger out–of–plane piezoelectricity. The exact values for their Fermi energy (*E_F_*), valence band maximum (VBM), conduction band minimum (CBM), and vacuum level (*V_L_*) are listed in Table 2. As shown in Figure 3d,e, X–PG is an indirect semiconductor under the PBE and HSE functions. Its VBM is located between the high symmetry points G (0, 0, 0) and X (0.5, 0, 0), and its CBM is located between the M (0.5, 0.5, 0) and G points, which is consistent with the situation for PG (see Figure S1a). The projection band shows that the Si (Ge) atom contributes more to the CBM than the VBM. The bandgaps of the Si–PG (0.78 eV) and Ge–PG (0.89 eV) monolayers under the HSE06 function are slightly larger than those under the PBE function and much smaller than that of PG (2.20 eV), by 2.82 and 2.47 times, respectively. It can be seen that the substitution of X atoms can regulate the bandgap of PG, which turns PG from a wide–gap semiconductor into a narrow–gap semiconductor. The work function Φ is used to evaluate an electron’s ability to escape from the surfaces of a material [37], which can be calculated as Φ = *V_L_* − *E_F_*. As shown in Table 2, the relationship between the work functions of these monolayers as calculated under the HSE06 function is Φ_Ge–PG_ (4.85 eV) < Φ_Si–PG_ (5.26 eV) < Φ_PG_ (6.68 eV), and the same applies under the PBE function. This means that the electrons inside Ge–PG escape most easily from this material’s surface among these monolayers. This corresponds to the piezoelectricity of Ge–PG being better than that of PG and Si–PG. In these monolayers, lower work function values are beneficial for enhancing piezoelectricity.

**Figure 4:**
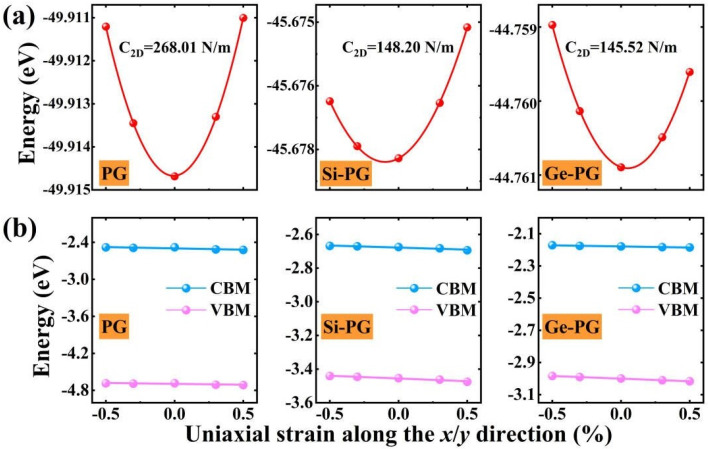
(**a**) The energy shifting and (**b**) band–edge positions as a function of the uniaxial strain in the *x* or *y* transport direction in PG and X–PG monolayers.

The in–plane carrier mobility of the X–PG monolayers is calculated through deformation potential approximation [38]. Since these monolayers have the same lattice constants in the *x* and *y* directions (*a* = *b*), the carrier transport characteristic in the *x* direction is equivalent to that in the *y* direction. As shown in Figure 4a,b, the energy shifting and band–edge positions of the PG and X–PG monolayers present parabolas and linear functions. As shown in Table 3, the hole mobility *μ_h_* of the Si–PG (4.31 × 10^3^ cm^2^ V^−1^ s^−1^) and Ge–PG (57.33 × 10^3^ cm^2^ V^−1^ s^−1^) monolayers is 28.73 and 382.20 times higher than that of PG (0.15 × 10^3^ cm^2^ V^−1^ s^−1^). This is mainly because the hole effective mass of the X–PG monolayers is smaller than that of PG, by 6.29 and 12.94 times, respectively. A lower carrier effective mass is beneficial for increasing the corresponding carrier mobility. On the contrary, the in–plane electron mobility of X–PG decreases with an increase in the electron effective mass compared with PG. *μ_h_* of Ge–PG is higher than that of other 2D materials with a high hole mobility, like graphene field–effect transistors (FETs) (9.21 × 10^3^ cm^2^ V^−1^ s^−1^) [39], MoTe_2_ passivated by Al_2_O_3_ (0.13 × 10^3^ cm^2^ V^−1^ s^−1^) [40], and ion–compensated WSe_2_ (0.10 × 10^3^ cm^2^ V^−1^ s^−1^) [41], by 6.22, 441.00, and 573.30 times, respectively. This further illustrates the extremely high hole mobility of the Ge–PG monolayer, which could be used in excellent high–speed electronic transmission devices such as high–frequency transistors and logic gates.

As shown in Table 4, PG has very high symmetry D_2d_ and thus does not show piezoelectricity in the 11 or 33 directions, and the corresponding piezoelectric coefficients are 0. The substitutions of the X atoms lead the X–PG monolayers to have the space point group C_1_ with the fewest symmetric operations. This causes them to exhibit strong piezoelectricity in the above directions. Among them, Ge–PG has the best piezoelectric performance, with the largest piezoelectric coefficients in each direction. The in–plane piezoelectric strain coefficient *d*_11_ value of Ge–PG (8.43 pm/V) is larger than that of the monolayers MoS_2_ (3.73 pm/V) [42], GaAs (1.50 pm/V) [43], CrSCl (0.80 pm/V) [44], and *h*–BN (0.60 pm/V) [45], by 2.26, 5.62, 10.54, and 14.05 times, respectively. The out–of–plane piezoelectric strain coefficient *d*_33_ value of Ge–PG (3.63 pm/V) is larger than that of 2D black phosphorus (2.24 pm/V) [46], Quasi–2D ZnO–NS (2.10 pm/V) [13], and 2D SnS_2_ (2.00 pm/V) [47], by 1.62, 1.73, and 1.82 times, respectively. The Ge–PG monolayer has excellent in–plane and out–of–plane piezoelectricity, and thus it has great potential to be applied as an electromechanical coupling nanodevice.

**Figure 5:**
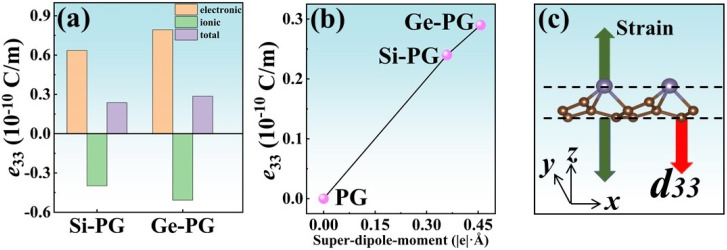
(**a**) Electron–contributed, ion–contributed, and total values of the out–of–plane piezoelectric stress coefficient *e*_33_ of X–PG monolayers. (**b**) The *e*_33_ value of PG and X–PG as a function of the super–dipole–moment inside them. (**c**) Schematic diagram of the out–of–plane piezoelectric strain coefficient *d*_33_ of Ge–PG.

The piezoelectric strain coefficient of a material is contributed to by both the ions and electrons of the atoms within it (see formula S1 in the Supplementary Materials). As shown in Figure 5a,b, the out–of–plane piezoelectric stress coefficients *e*_33_ of the X–PG monolayers are mainly contributed to by their electrons, and the polarizations generated by electrons and ions are opposite. This suggests that the electrons in these monolayers are more sensitive to external stress than the ions. According to formula S3, the piezoelectric strain coefficient *d_ik_* is derived from the coupling of the piezoelectric stress coefficient *e_il_* with the elastic properties of the material. Therefore, it is necessary to study the internal physical mechanism of *e_il_*. For PG and X–PG monolayers, the concepts of their positive and negative Born effective charge (BEC) tensors *h*(±) and the super–dipole–moment *P_S_* in the vertical direction (*z*–axis) can explain the difference between their *e*_33_ values well [48], which are expressed as

(4) $$h(+)=\frac{{\sum_{i}\left(Z_{33}^{*}A_{z}\right)}_{i}}{{\sum_{i}\left(Z_{33}^{*}\right)}_{i}},h(-)=\frac{{\sum_{j}\left(Z_{33}^{*}A_{z}\right)}_{j}}{{\sum_{j}\left(Z_{33}^{*}\right)}_{j}},$$

(5) $$P_{s}=\left|{\sum_{i}\left(Z_{33}^{*}\right)}_{i}\right|\times [h(+)-h(-)],$$

where *i* (*j*), *A_Z_*, and ${\sum_{i}\left(Z_{33}^{*}\right)}_{i}$ represent the atoms with positive (negative) *Z*_33_* BECs, the coordinates of the corresponding atom *A* along the *z*–axis, and the sum of all atomic positive *Z*_33_* values (the total positive *Z*_33_* values), respectively. The super–dipole–moment concept contains the physical idea that the positive and negative BEC carried by each atom inside the material is equivalent to two spatial sites to construct the dipole moment inside the material. The polarization vector corresponding to this dipole moment (the super–dipole–moment) is *P_s_*, and its direction points from *h*(−) to *h*(+). As shown in Figure 5c, *e*_33_ of the PG and X–PG monolayers increases with the super–dipole–moment. This regulation is defined as the super–dipole–moment effect [49]. The exact data on the total positive *Z*_33_* values, the distance between the positive and negative *Z*_33_* BEC tensors, and the super–dipole–moment of these monolayers are listed in Table S1. The reason PG does not exhibit out–of–plane piezoelectricity is that its *h*(+) is equal to *h*(−). According to formula 5, PG’s super–dipole–moment is 0. The signs of *e*_33_ and the super–dipole–moment of the X–PG monolayers are all positive, which indicates that the positive BEC tensor in these monolayers is higher than the negative BEC tensor. Therefore, the super–dipole–moment can be used to directly determine the vertical polarization direction of these materials (see vector *P* shown in Figure 1). As shown in Figure 5c, taking Ge–PG, for instance, *d*_33_ of X–PG is reversed along the *z*–axis, which is opposite to *e*_33_. This is because of the coupling between their out–of–plane piezoelectric stress coefficients and elastic properties. 

## 4. Conclusions

In summary, X–PG is predicted to be a 2D narrow–gap semiconductor with extremely high in–plane hole mobility and excellent piezoelectricity. Since PG is a wide–gap semiconductor, this indicates that the substitution of X atoms has a regulatory effect on the bandgap of PG. The Ge–PG monolayer shows better performance in all aspects, and its *x* (*y*)–direction hole mobility, in–plane strain piezoelectric coefficient *d*_11_, and out–of–plane strain piezoelectric coefficient *d*_33_ reach up to 8.43 pm/V, 3.63 pm/V, and 57.33×10^3^ cm^2^ V^−1^ s^−1^, respectively. The differences in their bond angles and structural characteristics contribute to the Poisson’s ratio inversion between PG, Si–PG, and Ge–PG. In terms of the physical mechanism aspect, the super–dipole–moment effect exists in these monolayers. The out–of–plane stress piezoelectric coefficient *e*_33_ of PG and X–PG increases with the super–dipole–moment, and the direction of *e*_33_ is consistent with that of the super–dipole–moment. Therefore, X–PG is predicted to have prominent potential for nanodevices applied as electromechanical coupling systems, wearable ultra–thin devices, high–speed electronic transmission devices, and so on. 

## Figures and Tables

**Table 1:** Lattice constant *a* (=*b*), thickness (*h*) (in units of Å), elastic stiffness coefficient *C_kl_*, Young’s modulus *Y_a_* (=*Y_b_*) (in units of N/m), and Poisson’s ratio ν*_a_* (=ν*_b_*) of PG and X–PG monolayers.

Monolayer	*a*	*h*	*C*_11_	*C*_12_	*C*_66_	*Y_a_*	ν*_a_*
PG	3.64	1.21	269.08	−21.27	151.15	267.40	−0.079
Si–PG	3.75	1.91	185.60	−4.15	13.58	178.41	−0.001
Ge–PG	3.76	2.05	146.68	31.62	27.75	142.17	0.162

**Table 2:** Fermi energy (*E_F_*), VBM, CBM, vacuum level (*V_L_*), and work function (Φ) of PG and X–PG monolayers (in units of eV).

Monolayer		*E_F_*	VBM	CBM	*V_L_*	Φ
PG	PBE	−4.46	−4.68	−2.48	1.52	5.98
	HSE06	−5.14	−5.36	−2.09	1.53	6.68
Si–PG	PBE	−3.17	−3.41	−2.67	2.10	5.27
	HSE06	−3.24	−3.52	−2.66	2.02	5.26
Ge–PG	PBE	−2.65	−2.92	−2.10	2.11	4.76
	HSE06	−2.60	−2.94	−2.04	2.18	4.85

**Table 3:** In–plane stiffness (*C*_2*D*_), carrier’s effective mass (m*), deformation potential constant (*E_d_*), and mobility for electrons and holes (*μ*) in the *x* direction of PG and X–PG monolayers.

	*C*_2*D*_ (N/m)	Carrier Type	m* (m_e_)	*E_d_* (eV)	*μ* (cm^2^ V^−1^ s^−1^)
PG	268.01	Hole	2.20	−3.89	0.15 × 10^3^
		Electron	−1.09	−2.85	0.32 × 10^3^
Si–PG	148.20	Hole	0.35	−3.26	4.31 × 10^3^
		Electron	2.52	−2.45	0.05 × 10^3^
Ge–PG	145.52	Hole	0.17	−3.27	57.33 × 10^3^
		Electron	1.73	−1.37	0.10 × 10^3^

**Table 4:** The piezoelectric stress and strain coefficients *e_il_* (in units of 10^−10^ C/m) and *d_ik_* (in units of pm/V) of PG and X–PG monolayers.

Monolayer	*e*_11_	*e*_33_	*d*_11_	*d*_33_
PG	0	0	0	0
Si–PG	6.95	0.24	3.48	−2.05
Ge–PG	9.79	0.29	8.43	−3.63

## Data Availability

The data used to generate the curves shown are available on request.

## Supplementary Materials

The following supporting information can be downloaded at: https://www.mdpi.com/article/10.3390/nano14161358/s1, Figure S1: (a) Electronic band structure of PG calculated by HSE06 function. (b) Phono spectrum of PG.; Table S1: The total positive *Z_33_^*^* BECs $\underset{i}{\sum }{\left(Z_{33}^{*}\right)}_{i}$ (|e|), the distance between positive and negative *Z_33_^*^* BECs–center *h*(+)–*h*(–) (Å) and the BECs–dipole–moment *P_B_* (|e|·Å) of PG, Si–sub and Ge–sub PG monolayers. References [50,51] are cited in the supplementary materials.
