# Supplementary material for: Excellent Hole Mobility and Out–of–Plane Piezoelectricity in X–Penta–Graphene (X = Si or Ge) with Poisson’s Ratio Inversion
Source: Nanomaterials (Basel). 2024 Aug 17;14(16):1358. doi: 10.3390/nano14161358 (PMC11357275; doi:10.3390/nano14161358)
Supplement: Supplementary file 1 [file nanomaterials-14-01358-s001.zip › nanomaterials-3118193-supplementary.pdf]

Supplementary Material

Excellent hole mobility and out-of-plane piezoelectricity in X-penta-graphene (X = Si or Ge) with Poisson's ratio inversion

Si-Tong Liu[1,†], Xiao Shang[1,*,†], Xi-Zhe Liu,[1] Xiao-Chun Wang[2], Fu-Chun Liu,[1,*] Jun Zhang,[1,*]

[1] Institute of Atomic and Molecular Physics, Jilin University, Changchun 130012, People's Republic of China; sitong22@mails.jlu.edu.cn (S. L.); shangxiao22@mails.jlu.edu.cn (X. S.); liu_xizhe@jlu.edu.cn (X. L.); lfc@jlu.edu.cn (F.L.); junzhang@jlu.edu.cn (J. Z.)

[2] School of Physics Science and Information Technology, Liaocheng University, Liaocheng 252000, People's Republic of China; wangxiaochun@tsinghua.org.cn (X.W.)

[*] Correspondence: shangxiao22@mails.jlu.edu.cn, lfc@jlu.edu.cn and junzhang@jlu.edu.cn

[†]These authors contributed to the work equally and should be regarded as co-first authors.

Theory of piezoelectricity and deformation potential approximation in PG and X-sub PG monolayers

The piezoelectric stress coefficient is determined by combining the contributions from both ions and electrons:[1]

$$e_{ijk} = \frac{dP_i}{d\varepsilon_{jk}} = e_{ijk}^{ion} + e_{ijk}^{ele}, \quad \text{(S1)}$$

where ε_{jk} is the stress tensor and P_i is the intrinsic polarization tensor. i, j, and k represent the x, y, and z axes, respectively. Similarly, the piezoelectric strain coefficient d_{ijk} can be defined as the derivative of the P_i with respect to the strain tensor σ_{jk}:

$$d_{ijk} = \frac{dP_i}{d\sigma_{jk}}, \quad \text{(S2)}$$

For simplicity, in the condensed Voigt notation, the third-order tensors d_{ijk} and e_{ijk} are commonly denoted as d_{il} and e_{il}. Here, the subscript i corresponds to the x, y, or z axes, indicated by the numbers 1, 2, and 3. The subscript l represents the second-order tensor xx, yy, zz, yz, zx, xy, which are denoted by the numbers 1, 2, 3, 4, 5, and 6, respectively.[2] The fourth-order tensor elastic stiffness coefficient C_{kl} serves as a link connecting e_{il} and d_{ik}:

$$e_{il} = d_{ik}C_{kl}, \quad \text{(S3)}$$

where the subscript i in the piezoelectric strain coefficient indicates the polarization direction.

The e_{il}, d_{ik} and C_{kl} are all determined by the lattice symmetry, with each being expressed as a matrix. The PG monolayer possesses a D_{2d} space point group, allowing for the expression of C_{kl} in PG monolayers as follows:

$$\mathrm{e}_{il} = \begin{pmatrix} 0 & 0 & 0 & e_{14} & 0 & 0 \\ 0 & 0 & 0 & 0 & e_{25} & 0 \\ 0 & 0 & 0 & 0 & 0 & e_{36} \end{pmatrix}, \quad \text{(S4)}$$

$$C_{kl} = \begin{pmatrix} C_{11} & C_{12} & C_{13} & 0 & 0 & 0 \\ C_{12} & C_{11} & C_{13} & 0 & 0 & 0 \\ C_{13} & C_{13} & C_{33} & 0 & 0 & 0 \\ 0 & 0 & 0 & C_{44} & 0 & 0 \\ 0 & 0 & 0 & 0 & C_{55} & 0 \\ 0 & 0 & 0 & 0 & 0 & C_{66} \end{pmatrix}, \tag{S5}$$

$$d_{ik} = \begin{pmatrix} 0 & 0 & d_{13} & d_{14} & d_{15} & 0 \\ 0 & 0 & d_{23} & d_{24} & d_{25} & 0 \\ 0 & 0 & 0 & 0 & 0 & d_{36} \end{pmatrix}, \tag{S6}$$

The Si-sub and Ge-sub PG monolayers have space point group C_1, which manifests that the symmetry of them is extremely low. There is nearly uncorrelated between the different matrix elements in the matrices e_{il}, d_{ik} and C_{kl} of Si-sub and Ge-sub PG monolayers, thus these matrices will not be showed here.

The relational expressions for the out-of-plane piezoelectric coefficients are derived:

$$d_{31} = \frac{Ae_{31} + Be_{32} + Ce_{33}}{F}, \tag{S7}$$

$$d_{33} = \frac{Ce_{31} + De_{32} + Ee_{33}}{F}, \tag{S8}$$

where $A = C_{22}C_{33} - C_{23}^2$, $B = C_{13}C_{23} - C_{12}C_{33}$, $C = C_{12}C_{23} - C_{13}C_{22}$, $D = C_{12}C_{13} - C_{11}C_{23}$, $E = C_{11}C_{22} - C_{12}^2$ and $F = C_{12}(C_{13}C_{23} - C_{12}C_{33}) + C_{22}(C_{11}C_{33} - C_{13}^2) + C_{23}(C_{13}C_{12} - C_{11}C_{23})$. Since the lattice constants of PG, Si-sub, Ge-sub PG monolayers are equal in the x and y directions (a=b), their piezoelectric coefficients in the 31 and 32 directions are equal. Then the formulas S7 and S8 can be further written as:

$$d_{31} = \frac{(A+B)e_{31} + Ce_{33}}{F}, \tag{S9}$$

$$d_{33} = \frac{(C+D)e_{31} + Ee_{33}}{F}, \tag{S10}$$

The deformation potential approximation is a widely used method for determining carrier mobility.[3-5] In the case of 2D structures, the calculation of carrier mobility follows a specific formula:[6]

$$\mu_{2D} = \frac{e\hbar^3 C_{2D}}{k_B T m^* \sqrt{m_x m_y} E_d^2}, \tag{S11}$$

where e is the elementary charge, C_{2D} denotes for the elastic modulus, E_d signifies the deformation potential constant, k_B and $\hbar$ correspond to the Boltzmann constant and the reduced Planck constant, m^* represents for effective mass and the average effective mass of carriers, and T = 300 K is the temperature utilized in these calculations.

The effective masses of electrons (m_e^*) and holes (m_h^*) can be determined by fitting a parabolic function to the band edges (CBM and VBM) using the following expression:

$$\frac{1}{m^*} = \frac{1}{\hbar}\left|\frac{\partial^2 E(k)}{\partial k^2}\right|, \tag{S12}$$

where $E(k)$ represents the energy dependence on the wave vector k at the CBM/VBM in the k_x-k_y plane. The carrier transport characteristics are studied in two distinct directions within the reciprocal lattice space.

The 2D materials' elastic modulus C_{2D} is expressed as following:

$$C_{2D} = \frac{1}{S_{uni}} \frac{\partial^2 E_{tot}}{\partial \varepsilon_{uni}^2}, \tag{S13}$$

where S_{uni}, E_{tot} and ε_{uni} symbolize the optimized unit cell area, total energy and uniaxial strain along the x and y carrier transport directions, respectively. The deformation potential constant E_d is determined using the formula:

$$E_d = \frac{\Delta E_{edge}}{\varepsilon_{uni}}, \tag{S14}$$

the term ΔE_{edge} denotes the energy shifting of the band edges relative to the vacuum level.

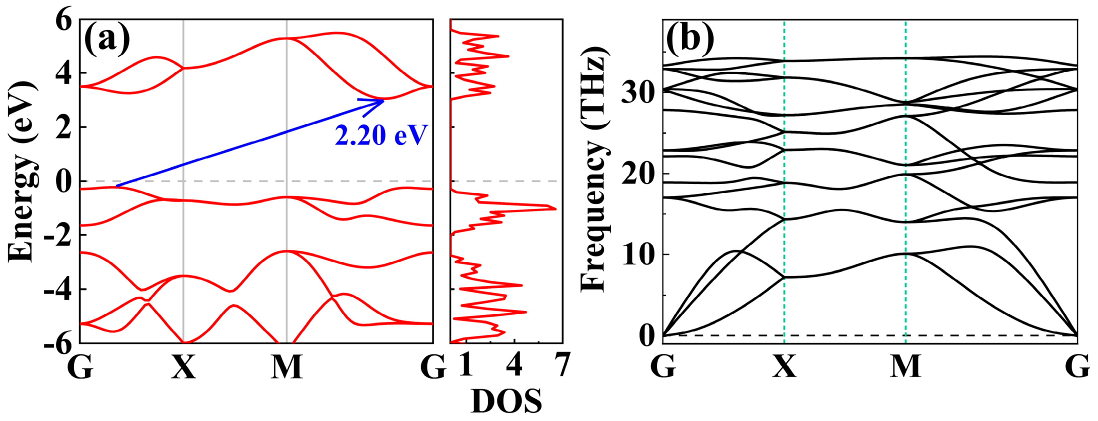

Figure S1. (a) Electronic band structure of PG calculated by HSE06 function. (b) Phono spectrum of PG.

Table S1. The total positive Z_{33}^* BECs $\sum_i(Z_{33}^*)_i$ (|e|), the distance between positive and negative Z_{33}^* BECs-center $h(+) - h(-)$ (Å) and the BECs-dipole-moment P_B (|e|·Å) of PG, Si-sub and Ge-sub PG monolayers.

Material	$\sum_i(Z_{33}^*)_i$	$h(+) - h(-)$	P_B
PG	0.25	0	0
Si-sub	0.29	1.24	0.36
Ge-sub	0.32	1.43	0.46

References

1. Dong, Liang, Jun Lou, and Vivek B. Shenoy. "Large in-Plane and Vertical Piezoelectricity in Janus Transition Metal Dichalchogenides." *Acs Nano* 11 (2017): 8242-48.
2. Zhang, He-Na, Yang Wu, Chunhua Yang, Liang-Hui Zhu, and Xiao-Chun Wang. "Enhanced out-of-Plane Piezoelectricity of Group-Iii(a) Janus Hydrofluoride Monolayers." *Physical Review B* 104 (2021): 235437.
3. Bardeen, John, and William Shockley. "Deformation Potentials and Mobilities in Non-Polar Crystals." *Physical Review* 80 (1950): 72-80.
4. Hieu, N. N., H. V. Phuc, A. I. Kartamyshev, and T. V. Vu. "Structural, Electronic, and Transport Properties of Quintuple Atomic Janus Monolayers Ga_2sx_2 (X=

O, S, Se, Te): First-Principles Predictions." *Physical Review B* 105 (2022):075402.

5. Zhang, T., Y. D. Ma, L. Yu, B. B. Huang, and Y. Dai. "Direction-Control of Anisotropic Electronic Behaviors *Via* Ferroelasticity in Two-Dimensional α-MPI (M = Zr, Hf)." *Materials Horizons* 6 (2019): 1930-37.

6. Wan, W. H., S. Zhao, Y. F. Ge, and Y. Liu. "Phonon and Electron Transport in Janus Monolayers Based on Inse." *Journal of Physics-Condensed Matter* 31 (2019):435501.
